# Tidal Volume Estimation Using the Blanket Fractal Dimension of the Tracheal Sounds Acquired by Smartphone

**DOI:** 10.3390/s150509773

**Published:** 2015-04-27

**Authors:** Natasa Reljin, Bersain A. Reyes, Ki H. Chon

**Affiliations:** Department of Biomedical Engineering, University of Connecticut, 260 Glenbrook Road, Storrs, CT 06269, USA; E-Mails: bersain.reyes@uconn.edu (B.A.R.); kchon@engr.uconn.edu (K.H.C.)

**Keywords:** blanket fractal dimension, tidal volume, tracheal sounds, smartphone

## Abstract

In this paper, we propose the use of blanket fractal dimension (BFD) to estimate the tidal volume from smartphone-acquired tracheal sounds. We collected tracheal sounds with a Samsung Galaxy S4 smartphone, from five (*N* = 5) healthy volunteers. Each volunteer performed the experiment six times; first to obtain linear and exponential fitting models, and then to fit new data onto the existing models. Thus, the total number of recordings was 30. The estimated volumes were compared to the true values, obtained with a Respitrace system, which was considered as a reference. Since Shannon entropy (SE) is frequently used as a feature in tracheal sound analyses, we estimated the tidal volume from the same sounds by using SE as well. The evaluation of the performed estimation, using BFD and SE methods, was quantified by the normalized root-mean-squared error (NRMSE). The results show that the BFD outperformed the SE (at least twice smaller NRMSE was obtained). The smallest NRMSE error of 15.877% ± 9.246% (mean ± standard deviation) was obtained with the BFD and exponential model. In addition, it was shown that the fitting curves calculated during the first day of experiments could be successfully used for at least the five following days.

## 1. Introduction

Tracheal sounds are defined as those that are detected or heard over the extrathoracic part of the trachea [1]. Tracheal sounds are strong, and cover a wide frequency range [2]. As part of respiratory sounds, they play an important role in monitoring respiratory activity, as well as in detection of pulmonary diseases [1,2,3].

Respiratory activity is one of the vital signs, and as such requires an adequate attention. Tidal volume is one of the parameters for monitoring respiratory activity [4]. It plays an important role for both healthy people and people with respiratory diseases, hence measuring and checking volume’s values can be helpful, especially in assessing risky situations involving respiratory failure [4,5,6]. Tidal volume is defined as the volume of air exchanged in one breath, and is commonly measured at the mouth [1,2,7]. The average value is about 500 mL per breath at rest [2,7]. Various methods exist for measuring the tidal volume, such as spirometry, whole-body plethysmography, inductance plethysmography, and electrocardiography [2,8,9,10]. However, these methods require the use of specialized equipment, and cannot be easily applied in nonclinical settings. Therefore, there is a need for a miniature monitoring device that can be used in everyday situations and not only in clinical and/or research settings [11]. In addition, with an extensive growth of electronic devices and their computational capabilities, the development of portable tidal volume estimation systems is now possible [12].

Several efforts have been made in the research oriented towards the estimation of tidal volume. In [13], the authors estimated volume by optically tracking reflective markers in three dimensions. Petrovic *et al.* proposed a technique for measuring tidal volumes by using a single fiber-grating sensor [14], while in [15] the authors estimated the tidal volume using Doppler radar signals. Chen *et al.* estimated tidal volume from the energy of the tracheal sounds [6]. To the best of our knowledge, there are no studies exploring the possibility to estimate tidal volume directly from smartphone-acquired tracheal sounds.

Smartphones are widely used nowadays. They have fast microprocessors, large storage capacities and a lot of media capabilities. In addition, the mobility of the smartphones is making them more popular for usage outside the clinics or research facilities, when they can be used for measuring vital signs and health monitoring, as shown in some of the previous works of our research group [16,17,18].

In this paper, we propose the use of blanket fractal dimension (BFD) for estimating the tidal volume from tracheal sounds acquired by a commercially available Android smartphone. Tracheal sounds, as part of respiratory sounds, are non-stationary and stochastic signals [2,19]. Due to this fact, some past studies investigated and showed successful applications of fractal analysis on tracheal and lung sounds [20,21,22,23,24]. None of these efforts was concerned with the tidal volume estimation using fractal analysis. In this study, we explore the possibility to estimate tidal volume using BFD, which, to the best of our knowledge, was not used for respiratory sound analysis. The estimated volumes were compared to peak-to-peak volumes obtained from a Respitrace signal, which was considered as a reference. In addition, we estimated volumes by obtaining Shannon entropy (SE) from the same tracheal sounds, and compared them to reference volumes. For testing the proposed method and comparing it with SE method, we collected signals from healthy and non-smoker volunteers for six days, for a total of 30 recordings. As a figure of merit, the normalized root-mean-squared errors (NRMSEs) were calculated in both cases. Repeated experiments were performed to investigate if the models for fitting data obtained during the first day of collecting signals could be successfully used on the data from the remaining days.

## 2. Materials and Methods

### 2.1. Subjects

Five healthy non-smoker volunteers (four males and one female), with the mean age and standard deviation of 27 ± 7.5 years, weight of 63.5 ± 5 kg, and height 173.2 ± 8.4 cm, were asked to participate in this study. Individuals with previous pneumothorax, chronic respiratory illnesses, and common cold were excluded from the study. This group of participants consisted of students and staff members from the University of Connecticut (UConn, Storrs, CT, USA). All participants signed a consent form approved by the Institutional Review Board of UConn.

### 2.2. Equipment and Acquisition of the Signals

In this study, two signals were acquired simultaneously: tracheal sounds and Respitrace signal. The tracheal sounds were collected using an acoustical sensor, which contained a subminiature electret microphone BT-21759-000 (Knowles Electronics, Itasca, IL, USA) placed in a plastic bell, which consisted of a conical coupler chamber [25], in accordance to previous findings [26]. The importance of this shape is that it provides an efficient transducer of air pressure fluctuations from the skin over the trachea to the microphone [27]. The acoustic sensor used in this study was developed by our colleagues at the Metropolitan Autonomous University at Mexico City, Mexico, and have been successfully applied for respiratory sound acquisitions [18,25,28]. The acoustic sensor was connected to the audio jack of the Samsung Galaxy S4 smartphone (Samsung Electronics Co., Seoul, Korea). The tracheal sounds were recorded using the built-in audio recorder application (Voice Recorder), with 16-bit per sample and 44.1 kHz sampling rate, and saved in the .wav format. Afterwards, the recorded files were transferred to a personal computer and processed offline using Matlab (R2012a, The Mathworks, Inc., Natick, MA, USA).

The Respitrace (nowadays known as Inductotrace) signal was obtained simultaneously with the tracheal sounds, from two Respibands (Ambulatory Monitoring, Inc., Ardsley, NY, USA), placed over the rib cage and abdomen. Respibands’ signals were digitized using 16-bit A/D converter (PowerLab/4SP, ADInstruments, Inc., Dunedin, New Zealand) at 10 kHz sampling rate, using the manufacturer’s software (LabChart 7, ADInstruments, Inc.). Prior to every participant’s recording, the Respibands were calibrated using a spirometer system (FE141 Spirometer, ADInstruments, Inc.) following the manufacturer’s manual, and the corresponding signal was considered as the reference for volume estimation. Calibration errors between Respibands and spirometer were obtained for every recording, and were less than 10%, which is in accordance to the manufacturer’s manual.

Experiments were performed in a regular dry lab which was held quiet. Respibands were placed over the participant’s rib cage and abdomen, while the acoustical sensor was fixed at the suprasternal notch using a double-sided adhesive ring (BIOPAC Systems, Goleta, CA, USA). The experiment consisted of three stages, and all were performed in standing posture:

1. Participants were asked to breathe through an 800 mL Spirobag (Ambulatory Monitoring, Inc., Ardsley, NY, USA) for about six respiratory cycles;
2. Participants were asked to follow a maneuver that consisted of increasing tidal volumes and then decreasing with each breath, ranging from participant’s comfortable lowest to highest volume, while breathing through a paper tube (tube’s length: 20 cm, internal diameter: 1.5 cm, external diameter: 2 cm), for approximately 2 min;
3. Participants were asked to repeat the same maneuver as in the second stage while breathing without the tube.

In everyday situations people do not have access to spirometers or Respibands, and the lack of portable and easily accessible device with possibility to control and limit the tidal volume is needed. Thus, in this research, we use a Spirobag, since it is easy to find and carry, and has an almost fixed volume (800 mL). The exact volume of the bag changes at each volunteers’ breathe. Hence, we used the Respitrace system as reference in order to know this volume, since the use of spirometer with a bag was practically prohibited in the experimental setup.

Since breathing through a tube adds some resistance to the respiratory tract and changes the natural way of breathing, one of the objectives was to investigate if this apparatus influences the estimation results. This was the reason for recording the third stage of the experiment.

**Figure 1:**
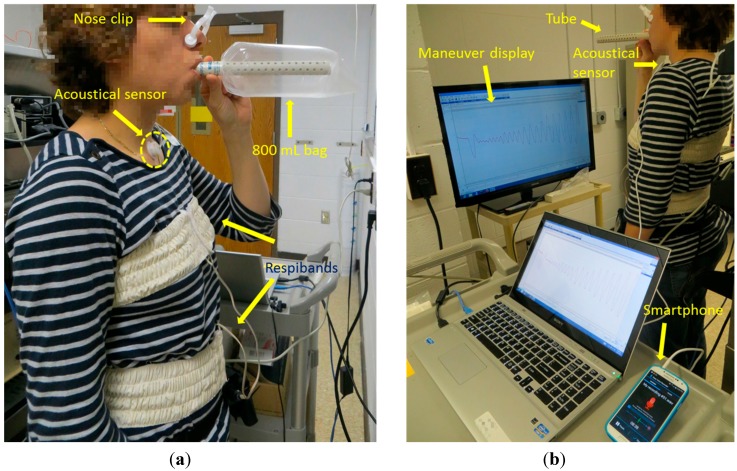
Simultaneous recordings of the tracheal sound (using a smartphone) and the volume signal (using Respibands). (**a**) The participant is breathing through 800 mL bag; (**b**) The participant is breathing through a tube while performing the respiratory maneuver.

In all three stages, initial and final apnea phases of approximately 5 s were acquired for automatic alignment purposes between the two recordings, as well as for recording the ambient noise levels. In the last two stages, after the initial apnea, participants were instructed to take a forced respiration cycle before performing the maneuver. In order to provide the visual feedback during the second and the third stage, the volume signal was displayed on a 40” monitor, placed in front of the participant. During the experiment, nose clips (MLA1008, ADInstruments, Inc.) were used to clamp the nostrils. An example of the set-up of the experiment is shown in Figure 1. Figure 1a depicts the first stage of the experiment, when the 800 mL bag was used, while Figure 1b shows the breathing maneuver through a tube (the second stage of the experiment).

### 2.3. Data Processing

**Figure 2:**
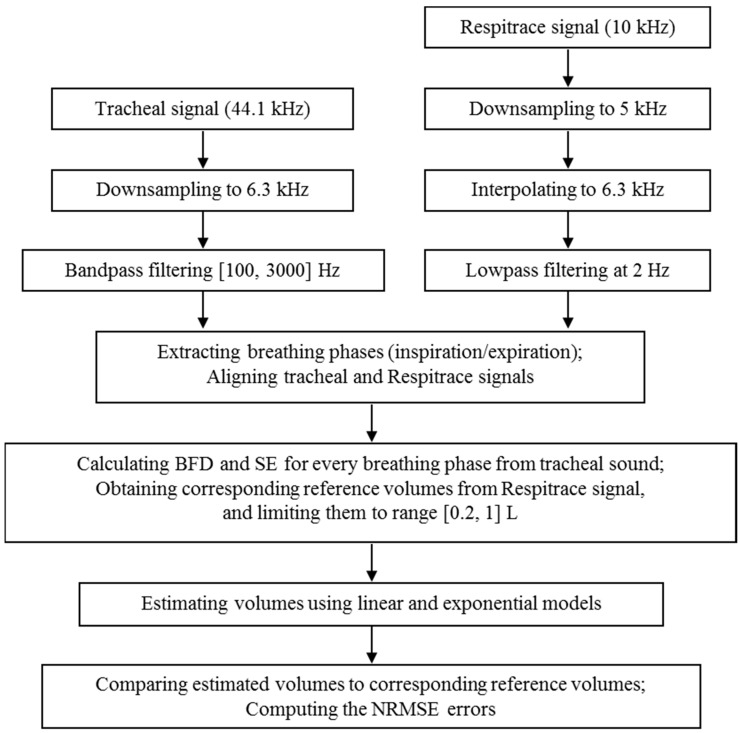
The flowchart showing the steps for tracheal sounds’ and Respitrace signal’s processing.

Figure 2 shows the flowchart of the data processing steps. The acquired tracheal sounds were first downsampled from 44.1 kHz to 6.3 kHz, and then digitally filtered with a 4th order bandpass Butterworth filter with cutoff frequencies 100 and 3000 Hz to minimize the effects of heart sounds and muscle interferences [27,29]. The volume signal was first downsampled from 10 kHz to 5 kHz, and then interpolated to 6.3 kHz in order to achieve the same sampling frequency as the tracheal sounds. Lastly, the volume signal was lowpass filtered at 2 Hz with a 4th order Butterworth filter.

**Figure 3:**
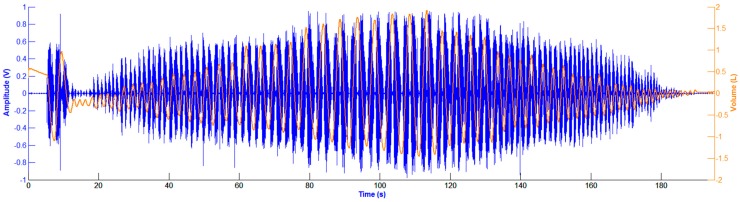
Filtered, detrended and aligned tracheal sounds and volume signal during the respiratory maneuver. Tracheal sound (in volts) is represented in blue, while volume signal (in liters) is in orange.

The automatic extraction of the breathing phases (inspiration/expiration) was performed from the volume signal, by finding its corresponding local maxima and minima during the respiratory maneuver and computing the slope of the volume at each phase [18]. The tracheal sounds and the volume signal were recorded simultaneously, however, due to the different times of pressing the start buttons, the two signals were aligned manually. Figure 3 depicts an example of the filtered, detrended and aligned tracheal sounds and volume signal during the respiratory maneuver.

The volume signal, acquired with the Respibands, was assumed as the reference. For every breathing phase, the absolute volume difference between two consecutive extrema from the volume signal was calculated, and was considered as the true tidal volume value, *V_T_*. Two features were used for estimating the tidal volume from the tracheal sounds acquired by smartphone: blanket fractal dimension (BFD) and the integral of the Shannon entropy (SE). Every breathing phase (inspiration/expiration) from the tracheal sound was represented with one BFD and one SE value. In order to estimate the volume from these features, linear and exponential fitting curves were used. The estimated volumes are defined with the following:

(1) $$\begin{array}{l} V_{est\_l}=a\cdot F+b\\ V_{est\_e}=c\cdot e^{d\cdot F} \end{array}$$

where *V_est_l_* and *V_est_e_* are the estimated volumes with linear and exponential models, respectively, *a*, *b*, *c* and *d* are coefficients, and *F* is the value of the BFD or SE feature computed from the tracheal sounds.

The last step in the data processing is the comparison of the estimated volumes to the corresponding reference volume values, and the evaluation of the performed estimation via computation of the normalized root-mean-squared error (NRMSE) defined as follows:

(2) $$\begin{array}{l} NRMSE=\frac{RMSE}{mean\left(V_{T}\right)}\cdot 100\%\\ RMSE=\sqrt{\frac{\sum_{i=1}^{N}{\left(V_{T}\left(i\right)-V_{est}\left(i\right)\right)}^{2}}{P}} \end{array}$$

where *V_T_* is the volume obtained from Respitrace, *V_est_* denotes the estimated volume, *i.e.*, *V_est_l_* or *V_est_e_*, and *P* is the number of breathing phases during the maneuver.

Shannon entropy is a measure of uncertainty or irregularity of a process [30]. It is one of the features frequently used for analysis of respiratory sounds, and has been successfully applied to airflow estimation in the field of tracheal sound analysis [31]. For a random signal with a probability density function (pdf), *p*, SE is defined as:

(3) $$SE\left(p\right)=-\sum_{i=1}^{M}p_{i}\cdot \log p_{i}$$

where *M* is the number of outcomes of the random variable with pdf *p*. In this study, pdf is estimated using the method of Parzen’s windows with a Gaussian kernel [32,33]. More details on this method can be found in [18,31]. In this study we were concerned with the tidal volume estimation rather than respiratory airflow, and based on the relationship between these two variables over time, the integral of the SE over each corresponding breathing phase was used as feature for tidal volume estimation.

### 2.4. Blanket Fractal Dimension

Fractals are defined as 'a set having the fractal dimension strictly greater than its integer dimension’, and are used to describe non-regular and non-stationary structures [34,35,36]. There are two types of fractals: natural and deterministic. Natural fractals are structures that could be found in the nature, such as lungs, while deterministic fractals are constructed artificially, by applying predetermined replicating rules (e.g., the Von Koch curve, the Cantor set) [36,37]. Fractal structures may be quantified by fractal dimension, which is a number (usually non-integer) expressing the manner in which the irregular structure replicates itself through different scales [36,37]. Among various fractal dimensions, in this study we used blanket fractal dimension (BFD). The BFD was initially proposed for estimating fractal dimension of digital images (2D signals) [38], and is further extended to 1D signals [39].

In the case of 1D signals, the set of points within maximal distance *ε* from a curve is considered. Therefore, a strip of width 2*ε* that surrounds the curve is observed [40]. Blanket method creates the strip around the signal, defined by the upper and lower limiting lines, defined as follows [39]:

(4) $$\begin{array}{l} u_{\varepsilon }\left(i\right)=\max\left\{u_{\varepsilon -1}\left(i\right)+1,\underset{\left|m-i\right|\leq 1}{\max}u_{\varepsilon -1}\left(m\right)\right\}\\ b_{\varepsilon }\left(i\right)=\min\left\{b_{\varepsilon -1}\left(i\right)-1,\underset{\left|m-i\right|\leq 1}{\min}b_{\varepsilon -1}\left(m\right)\right\}\\ u_{0}\left(i\right)=b_{0}\left(i\right)=x\left(i\right) \end{array}$$

where *x*(*i*) represents the observed 1D signal, $u_{\varepsilon }\left(i\right)$ and $b_{\varepsilon }\left(i\right)$ are the upper and lower lines, respectively, *i* is the current sample of the signal, *m* denotes samples within the window around the current sample of the signal, and *ε* is the predefined maximal distance of upper/lower line from the signal. As can be noted from Equation (4), the upper/lower line is always calculated for the three consecutive samples: *i* − 1, *i*, and *i* + 1.

The area of the strip between upper and lower lines is defined as:

(5) $$A_{\varepsilon }=\sum_{i}\left\{u_{\varepsilon }\left(i\right)-b_{\varepsilon }\left(i\right)\right\}$$

from which the length of the curve *x* can be estimated as [39]:

(6) $$L\left(\varepsilon \right)=\frac{A_{\varepsilon }-A_{\varepsilon -1}}{2}$$

On the other hand, the length of the curve follows the power law [36]:

(7) $$L\left(\varepsilon \right)=C\cdot \varepsilon^{1-D}$$

where *C* is the constant and *D* is the blanket fractal dimension (BFD). By combining Equations (6) and (7), and using the least square approximation, blanket fractal dimension is calculated.

## 3. Results

All five participants performed the experiments described in Section 2.2 six times in six distinct days, thus creating a database of 30 recordings. The data collected on the first day were used for obtaining the linear and exponential models, while the data from the remaining five days were used for testing the previously obtained models. Each breathing phase, inspiration and expiration, was analyzed separately.

The linear and exponential fitting curves were calculated only from the first stage of the experiment performed during the first day, using two and three points, respectively, when the participant was breathing through an 800 mL bag for about six respiratory cycles. BFD and SE features were calculated from the smartphone acquired tracheal sounds, while the reference volume values were obtained from the Respitrace signal. This was performed for every inspiratory and expiratory phase, as well as for the portion of the signal during the initial apnea (denoted as background). For the linear fitting curve, for both BFD and SE features, it was found, experimentally, that two points, *A* and *B*, with the following coordinates:

(8) $$\begin{array}{l} A=(x_{1},y_{1})=(\text{mean}(\text{feature values for 800 mL}),\text{mean}(\text{volumes for 800 mL}))\\ B=(x_{2},y_{2})=(\text{feature value of background for 800 mL},\text{volume of background for 800 mL}) \end{array}$$

are sufficient for determining the fitting line.

Similarly, for exponential fitting curves, we found empirically that three points are sufficient, as follows. When using BFD features, the three points (*C*, *D*, *E*) are:

(9) $$\begin{array}{l} C=(x_{3},y_{3})=(\text{mean}(\text{BFD for 800 mL}),\text{mean}(\text{volumes for 800 mL}))\\ D=\left(x_{4},y_{4}\right)=\left(0.8,0\right)\\ E=\left(x_{5},y_{5}\right)=\left(2,2\right) \end{array}$$

and with SE features (points *F*, *G*, *H*):

(10) $$\begin{array}{l} F=(x_{6},y_{6})=(\text{mean}(\text{SE for 800 mL}),\text{mean}(\text{volumes for 800 mL}))\\ G=\left(x_{7},y_{7}\right)=\left(0,0.2\right)\\ H=\left(x_{8},y_{8}\right)=\left(6,2\right) \end{array}$$

**Figure 4:**
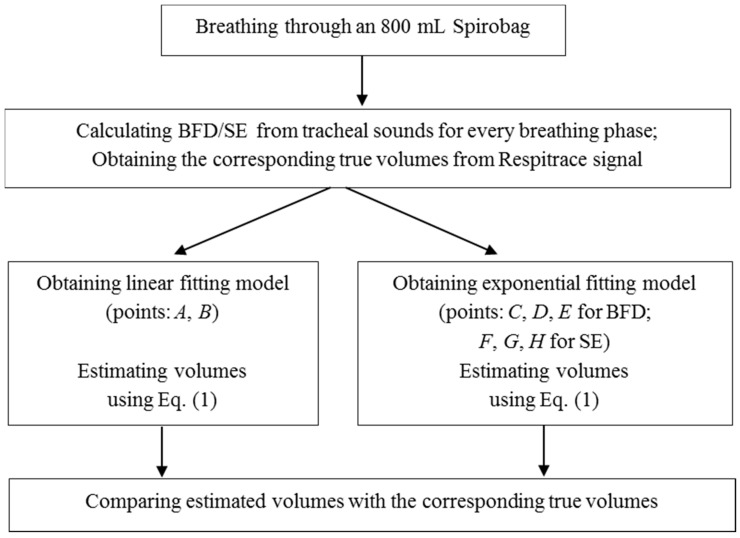
The flowchart showing the computation of the fitting models.

After investigating values of the BFD and SE features from all participants, we noticed that the upper limits were 2 and 6, for BFD and SE respectively. Therefore, we used these asymptotic values as abscissae of points *E* and *H*. Figure 4 illustrates the computation of the linear and exponential models.

After the linear and exponential curves are calculated, data from the second and the third stages of the experiment (breathing with and without a tube) were used to fit the curves, separately. BFD and SE features were calculated from the smartphone acquired tracheal sounds, and the corresponding volumes were estimated using Equation (1) for the linear and exponential models. Simultaneously, the true volume values were obtained from the reference Respitrace signal. Since the volume range for normal breathing is between 0.2 and 1 L [7], we limited the true volume values to this range, and used only the corresponding portions of tracheal sounds for analysis.

**Figure 5:**
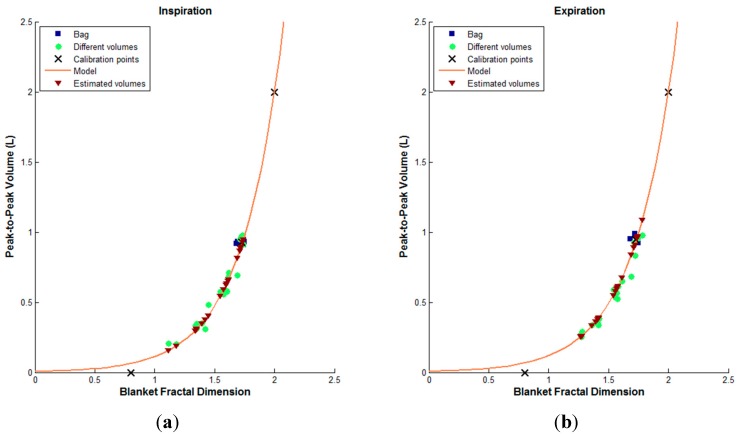
An example of the volume estimation from smartphone acquired tracheal sounds using BFD features and exponential model of one subject. The true volumes while breathing through a tube (green circles) are limited to a range from 0.2 to 1 L. (**a**) The inspiration phase; (**b**) The expiration phase.

An example of the volume estimation from smartphone acquired tracheal sounds using BFD features and exponential model, for both inspiration and expiration, of one subject is shown in Figure 5. The true tidal volume values (from Respitrace system) and their corresponding BFD values when breathing through 800 mL bag and tube are represented in blue squares and green circles, respectively, while the estimated volumes and their corresponding BFD features are depicted as brown triangles. The three points, shown as black marks in Figure 5 and given with Equation (9), are used for obtaining the exponential fitting curve, which is shown as a solid red curve.

**Figure 6:**
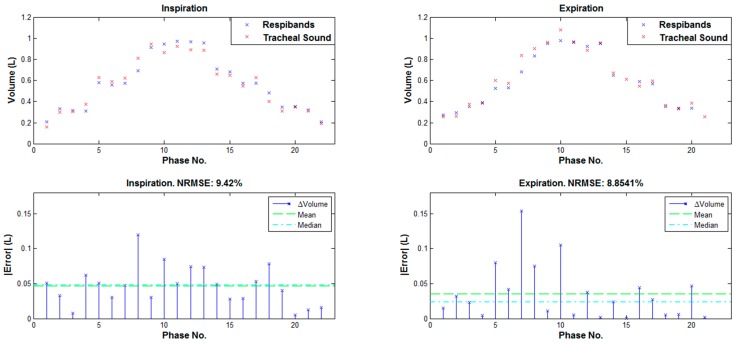
**Top:** Reference and estimated volumes for the same example as in Figure 5. **Bottom:** The corresponding NRMSE errors.

For every inspiration and expiration phase, when a true volume value was between 0.2 and 1 L, the estimated volumes were compared to their corresponding true volumes, and NRMSEs were calculated using Equation (2). In Figure 6 are shown the estimated and reference volumes, as well as the corresponding NRMSE errors for every inspiratory and expiratory phase for the same example as in Figure 5.

As can be noted from Figure 6, values of the volumes estimated from a smartphone acquired tracheal sounds using the BFD features are very similar to the volume values obtained from a Respitrace (reference) signal; and the NRMSE errors in both inspiration and expiration phases are low (less than 10%).

After the first day of experiments (later denoted as training), the participants repeated breathing maneuvers with and without a tube for five days (denoted as tests 1–5). The BFD and SE features were calculated from the tracheal sounds, and the volumes were estimated using the first day’s fitting curves. Simultaneously, the true volume values were obtained from the Respitrace signal. Again, the estimated volumes were compared to the true volumes, and NRMSEs were calculated.

In this study, we compared the volume estimation results when the proposed blanket fractal dimension is used as feature, with results obtained with Shannon entropy. Conditions of comparisons included: the type of the model (exponential, linear), the type of the apparatus (tube, no tube), and the breathing phase (inspiration, expiration). All combinations of conditions were made, and the corresponding ones were tested statistically, using the two-tailed paired *t*-tests (SPSS Statistics 20, IBM Corporation, Armonk, NY, USA). Table 1 contains the list of combinations and their corresponding *p*-values when statistically significant differences occurred (*p* < 0.05).

**Table 1:** Combinations of conditions when statistically significant differences were obtained, and their corresponding *p*-values. Results are grouped into 4 groups, based on the type of comparisons performed, *i.e.*, BFD *vs.* SE; inspiration *vs.* expiration; no tube *vs.* tube; exponential *vs.* linear model.

Type	Day	Conditions	*p*-value
BFD *vs.* SE	Test 4	Exponential, tube, inspiration	0.049
		Exponential, tube, expiration	0.015
		Exponential, no tube, expiration	0.011
		Linear, tube, inspiration	0.037
		Linear, tube, expiration	0.013
		Linear, no tube, expiration	0.002
	Test 5	Exponential, tube, expiration	0.017
		Linear, tube, expiration	0.006
		Linear, no tube, expiration	0.007
Inspiration *vs.* Expiration	Test 1	BFD, linear, tube	0.033
	Test 4	SE, linear, tube	0.025
	Test 5	BFD, linear, tube	0.022
		SE, exponential, tube	0.029
		SE, linear, tube	0.031
No tube *vs.* Tube	Training	SE, exponential, inspiration	0.016
	Test 4	BFD, linear, inspiration	0.042
	Test 5	BFD, linear, inspiration	0.033
Exponential *vs.* Linear	Training	BFD, tube, expiration	0.008
		BFD, no tube, expiration	0.038
	Test 4	BFD, tube, expiration	0.028
		SE, tube, expiration	0.018
	Test 5	SE, tube, expiration	0.028

**Figure 7:**
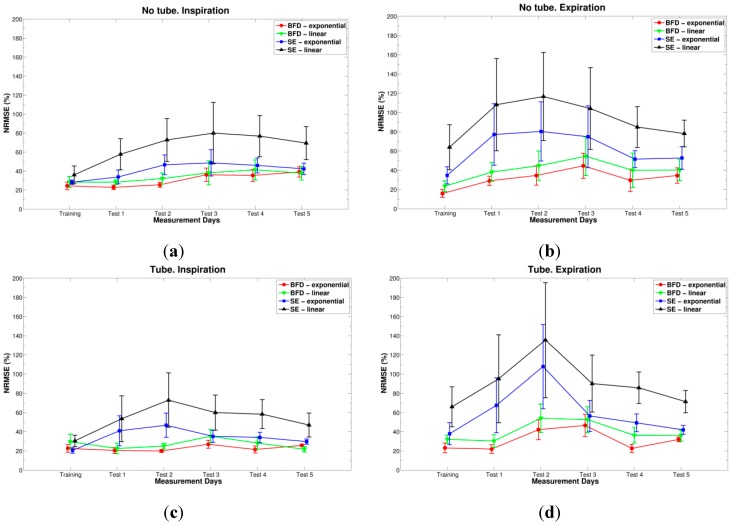
NRMSE errors (represented with its mean and standard error of the mean) when: BFD and exponential model (red circles), BFD and linear model (green downward triangles), SE and exponential model (blue squares), and SE and linear model (black triangles) are used. (**a**) No tube and inspiration; (**b**) No tube and expiration; (**c**) Tube and inspiration; (**d**) Tube and expiration.

In addition, for each combination, the comparisons between results (NRMSE errors) of the training day and the five test days were performed, and tested statistically using the repeated measures ANOVA with Bonferroni *post-hoc* tests (SPSS Statistics 20). The NRMSE errors are grouped into four parts, based on the apparatus and breathing phase, so that comparisons between features and models can be performed, and are depicted in Figure 7. These graphs show the changes in NRMSE errors throughout six days of experiments for all combinations of features and models simultaneously.

As can be concluded from the graphs in Figure 7, when blanket fractal dimension was used for volume estimation (red and green lines), the errors were lower at least two times than when Shannon entropy was used (blue and black lines), especially with the exponential model (red circles). Moreover, note that standard errors are also smaller when BFD is used. Statistically significant differences between the two features appeared during the fourth test day (for: exponential and linear models, with tube and both inspiration and expiration phases; and for both models, without tube and expiration) and the fifth test day (for: both models, with tube and expiration phase; and linear model, without a tube and expiration), as shown in Table 1.

**Figure 8:**
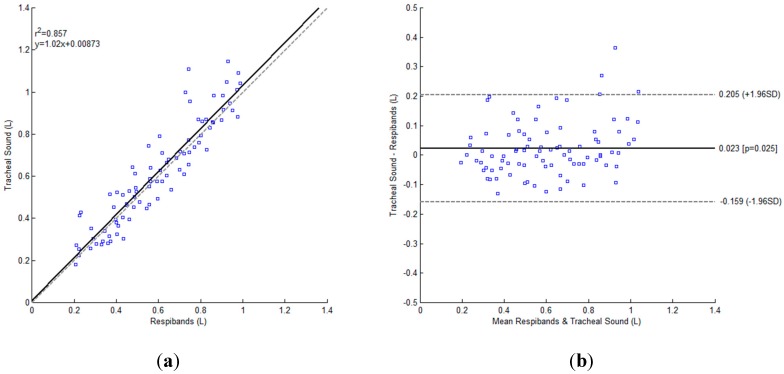
Bland-Altman plot for BFD feature with the exponential model, for expiratory phase, while the participants (*N* = 5) were breathing without a tube during the first day of experiments. (**a**) The regression plot: The unitary line is shown as gray dashed line, while the regression line is represented as black solid line; (**b**) Bland-Altman plot: The bias is represented as a solid black line and the 95% limits of agreement as gray dashed lines.

The smallest NRMSE error, with mean and standard deviation of 15.877% ± 9.246%, was obtained during the first day of experiments (training), when BFD feature with the exponential model was used, for expiratory phase, while the participants were breathing without a tube, Figure 7b. The Bland-Altman analysis showed a bias and standard deviation of 0.0226 ± 0.0918 L, and the corresponding results are presented in Figure 8.

By looking at the NRMSEs calculated for the remaining 5 days (test days), one can conclude that the smallest was always obtained with the BFD feature, exponential model and inspiration while breathing through a tube (errors ranging from 20% to 27%), Figure 7c, except for the fifth day, when linear model provided better estimation (error around 21%). No statistically significant differences were found between BFD exponential model from inspiratory and expiratory phases, as deduced from Table 1.

As was mentioned above, when BFD feature was used the errors were always smaller than with SE. In addition, one can conclude that the fitting curves obtained during the first day of experiments (training) can be successfully used for the following test days. This way, the participants do not need to perform all three stages of the experiments, and the fitting curves do not need to be calculated every day, as the previously determined could be used. In order to statistically compare errors throughout all six days of experiments, repeated measures ANOVA with Bonferroni *post-hoc* tests were performed, and was determined that there were no statistically significant differences between the days of experiments when BFD or SE was used as feature. According to Table 1, for the BFD using exponential model, no statistically significant differences were found between breathing through the tube or not.

## 4. Discussions and Conclusions

The goal of this study was to estimate tidal volume from the smartphone acquired tracheal sounds. The main challenge was to find a suitable feature to describe these sounds, such that the volume could be estimated directly from the sounds as accurate as possible. Respiratory sounds, and hence tracheal sounds, are non-stationary and stochastic signals [2], and as such they are suitable for fractal analysis [36]. We tested several ways for estimating fractal dimension, and decided to use the blanket fractal dimension because it was more suitable for describing and following the dynamics of the tracheal sounds, which was evident after exploring the results. Possible explanation could be the definition of the blanket fractal dimension itself. Blanket method creates a strip around the tracheal signal, closely following the changes in the signal. As the signal changes faster, the value of blanket fractal dimension becomes higher. In some past studies fractal analysis and fractal dimensions were used for analyzing tracheal and lung sounds [20,21,22,23,24]. Moreover, blanket fractal dimension was not used in respiratory sound analysis yet, and especially not for estimating the tidal volume, which are some of the novelties of this manuscript. In addition, to the best of our knowledge, none of the studies on tidal volume estimation has reported results based on tracheal sounds acquired by a smartphone.

In addition to BFD features, we used Shannon entropy (SE), as it is one of the features frequently used for analysis of respiratory sounds. In [41], the authors proposed a method to estimate airflow from tracheal sounds using SE. In [42], the authors proposed tidal volume estimation method by integrating airflow derived from tracheal sounds, which takes advantage of airflow/sound intensity relationship. As can be noted, the straightforward comparison between our method and method used in [42] is difficult to perform, since the conditions are not exactly the same. We estimated the tidal volume directly from tracheal sounds, using BFD as a feature, while Que *et al.* [42] obtained first the relationship between sounds’ amplitude and airflow, and then the volume by integrating the flow. Consequently, according to the provided results, the range of volume values in [42] was roughly between 0.3 and 0.8 L, while we limited volumes to a broader range [0.2, 1] L. That being said, the Bland-Altman analysis results of [42] were 0.009 ± 0.046 L (bias ± SD), while we found a bias and standard deviation of 0.0226 ± 0.0918 L. Chen *et al.* estimated tidal volume from the energy of the tracheal sounds [6]. The comparison of our results with those reported in [6] is not easy to perform since they are reported separately for each individual participant. If we compute the average results from the provided individually-based values reported in Table 1 [6]), we can conclude that the results are comparable. The volumes ranged from 0.15 to 0.5 L in [6], which is notably smaller range than the one used in this study. Note that in contrast to these two studies, the only external information needed to compute the calibration model with our proposed method was obtained with a simple bag at a known fixed value and not from a spirometer-like device.

After volumes were estimated from the smartphone acquired tracheal sounds, they were compared to the true volume values, obtained from Respitrace signal, which was considered as a reference in this study. The Respitrace signal was calibrated against the spirometer signal prior every recording and the obtained calibration errors were less than 10%, which is in accordance to the manufacturer’s manual. These reference volumes were limited to a range from 0.2 to 1 L, as it is the normal breathing range [7]. Inspiratory and expiratory phases were analyzed separately. Two fitting models, exponential and linear, were used for estimation. Our results indicate that the best estimation was obtained using blanket fractal dimension with exponential model, during expiratory phase, while participants were breathing without a tube, when the NRMSE error was 15.877% ± 9.246% (expressed as mean ± standard deviation). In addition, when the BFD is used as a feature, the NRMSEs were always smaller, at least twice, compared to the SE.

The experiments involved acquisition during six days. Data from the first day of experiments were used to construct estimation models, while the data from the remaining five days were plotted against the obtained models. The results show the possibility to successfully apply previously obtained fitting curves and to monitor tidal volume for at least five days. This way we introduce an easy calibration procedure, where there is no need to calculate fitting curves prior every consecutive experiment. In our future work, we plan to determine for how many days the existing models can be used.

This is a preliminary study, with the objective to estimate tidal volume in healthy participants, and not in patients with pulmonary diseases. Therefore, it was performed on five healthy participants, and for the future work we plan to expand the group. This study was limited to acquisition of tracheal sounds in standing posture without head movements. We expect that the results obtained with the proposed methodology would be in agreement with the study reported in [42], where the effects of body movements and posture changes on tidal volume estimates were investigated. Accordingly, we foresee that head movements without neck extension will not modify the obtained results and we do not anticipate an increase in estimation errors when moving to seated posture, but we do when moving from standing to supine posture, where a new calibration in latter posture would be required. It is worth to mention that all recordings were made in a regular dry lab, that was held quiet, and not in a special soundproof environment, hence making it applicable to real-life situations. Since spirometer is not a portable device, not easily accessed and fixed values of tidal volumes are hard to control, which results in additional turbulences and changes in breathing patterns, we used a Spirobag in order to obtain information at a known volume which in turn was employed in the estimation model. In addition, due to high performance capabilities of smartphones, by connecting an adequate acoustical sensor to a smartphone and using a Spirobag, a portable system for tidal volume estimation can be obtained.

In summary, in this manuscript we proposed a novel technique for estimation of tidal volume directly from the blanket fractal dimension of the tracheal sounds. The proposed method provided promising results and outperformed a method based on the Shannon entropy, which is frequently used in tracheal sounds analysis. Furthermore, we introduced an easy calibration procedure that does not require specialized devices and when combined with the proposed signal processing technique allows reasonable estimation for at least five days, which makes this method easier to use in everyday situations. The employment of smartphone-acquired tracheal sounds was also introduced for all of the above mentioned purposes. We foresee that similar efforts to the one presented here represent a step forward to the development of a mobile breathing monitoring system easily available for the general population.

## References

[1] Sovijarvi A.R.A., Dalmasso F., Vanderschoot J., Malmberg L.P., Righini G., Stoneman S.A.T. (2000). Definition of terms for applications of respiratory sounds. Eur. Respir. Rev..

[2] Moussavi Z. (2006). Fundamentals of Respiratory Sounds and Analysis.

[3] Sovijarvi A.R.A., Malmberg L.P., Charbonneau G., Vanderschoot J., Dalmasso F., Sacco C., Rossi M., Earis J.E. (2000). Characteristics of breath sounds and adventitious respiratory sounds. Eur. Respir. Rev..

[4] Folke M., Cernerud L., Ekström M., Hök B. (2003). Critical review of non-invasive respiratory monitoring in medical care. Med. Biol. Eng. Comput..

[5] Kuratomi Y., Okazaki N., Ishihara T., Arai T., Kira S. (1985). Variability of breath-by-breath tidal volume and its characteristics in normal and diseased subjects. Jpn. J. Med..

[6] Chen G., de la Cruz I., Rodriguez-Villegas E. Automatic lung tidal volumes estimation from tracheal sounds. Proceedings of the 36th Annual International Conference of the IEEE Engineering in Medicine and Biology Society (EMBC).

[7] Sherwood L. (2012). Fundamentals of Human Physiology.

[8] Semmes B.J., Tobin M.J., Snyder J.V., Grenvik A. (1985). Subjective and objective measurement of tidal volume in critically ill patients. Chest.

[9] Grossman P., Spoerle M., Wilhelm F.H. (2006). Reliability of respiratory tidal volume estimation by means of ambulatory inductive plethysmography. Biomed. Sci. Instrum..

[10] Sayadi O., Weiss E.H., Merchant F.M., Puppala D., Armoundas A.A. (2014). An optimized method for estimating the tidal volume from intracardiac or body surface electrocardiographic signals: Implications for estimating minute ventilation. Am. J. Physiol. Heart Circ. Physiol..

[11] Corbishley P., Rodriguez-Villegas E. (2008). Breathing detection: Towards a miniaturized, wearable, battery-operated monitoring system. IEEE Trans. Biomed. Eng..

[12] Earis J.E., Cheetham B.M.G. (2000). Future perspectives for respiratory sound research. Eur. Respir. Rev..

[13] Cala S.J., Kenyon C.M., Ferrigno G., Carnevali P., Aliverti A., Pedotti A., Macklem P.T., Rochester D.F. (1996). Chest wall and lung volume estimation by optical reflectance motion analysis. J. Appl. Physiol..

[14] Petrovic M.D., Petrovic J., Danicic A., Vukcevic M., Bojovic B., Hadzievski Lj., Allsop T., Lloyd G., Webb D.J. (2014). Non-invasive respiratory monitoring using long-period fiber grating sensors. Biomed. Opt. Express.

[15] Lee Y.S., Pathirana P.N., Steinfort C.L., Caelli T. (2014). Monitoring and analysis of respiratory patterns using microwave doppler radar. IEEE J. Transl. Eng. Health Med..

[16] Scully C.G., Lee J., Meyer J., Gorbach A.M., Granquist-Fraser D., Mendelson Y., Chon K.H. (2012). Physiological parameter monitoring from optical recordings with a mobile phone. IEEE Trans. Biomed. Eng..

[17] Lee J., Reyes B.A., McManus D.D., Mathias O., Chon K.H. (2013). Atrial fibrillation detection using an iPhone 4S. IEEE Trans. Biomed. Eng..

[18] Reyes B.A., Reljin N., Chon K.H. (2014). Tracheal sounds acquisition using smartphones. Sensors.

[19] Ahlstrom C., Johansson A., Hult P., Ask P. (2006). Chaotic dynamics of respiratory sounds. Chaos Solitons Fractals.

[20] Yap Y.L., Moussavi Z. Respiratory onset detection using variance fractal dimension. Proceedings of 23rd Annual International Conference of the IEEE Engineering in Medicine and Biology Society.

[21] Gnitecki J., Moussavi Z. Variance fractal dimension trajectory as a tool for heart sound localization in lung sounds recordings. Proceedings of 25th Annual International Conference of the IEEE Engineering in Medicine and Biology Society.

[22] Hadjileontiadis L.J., Rekanos I.T. (2003). Detection of explosive lung and bowel sounds by means of fractal dimension. IEEE Signal Process. Lett..

[23] Gnitecki J., Moussavi Z. (2005). The fractality of lung sounds: A comparison of three waveform fractal dimension algorithms. Chaos Solitons Fractals.

[24] Hadjileontiadis L.J. (2007). A novel technique for denoising explosive lung sounds: Empirical mode decomposition and fractal dimension filter. IEEE Eng. Med. Biol. Mag..

[25] Charleston-Villalobos S., Albuerne-Sanchez L., Gonzalez-Camarena R., Mejia-Avila M., Carrillo-Rodriguez G., Aljama-Corrales T. (2013). Linear and nonlinear analysis of base lung sound in extrinsic allergic alveolitis patients in comparison to healthy subjects. Methods Inf. Med..

[26] Druzgalski C.K., Donnerberg R.L., Campbell R.M. (1980). Techniques of recording respiratory sounds. J. Clin. Eng..

[27] Pasterkamp H., Kraman S.S., Wodicka G.R. (1997). Respiratory sounds: Advances beyond the stethoscope. Am. J. Respir. Crit. Care Med..

[28] Charleston-Villalobos S., Martinez-Hernandez G., Gonzalez-Camarena R., Chi-Lem G., Carrillo J.G., Aljama-Corrales T. (2011). Assessment of multichannel lung sounds parameterization for two-class classification in interstitial lung disease patients. Comput. Biol. Med..

[29] Reichert S., Gass R., Brandt C., Andres E. (2008). Analysis of respiratory sounds: State of the art. Clin. Med. Insights Circ. Respir. Pulm. Med..

[30] Papoulis A., Pillai S.U. (2002). Probability, Random Variables, and Stochastic Processes.

[31] Yadollahi A., Moussavi Z.M.K. (2006). A robust method for heart sounds localization using lung sounds entropy. IEEE Trans. Biomed. Eng..

[32] Parzen E. (1962). On estimation of a probability density function and mode. Ann. Math. Stat..

[33] Duda R.O., Hart P.E., Stork D.G. (2000). Pattern Classification.

[34] Mandelbrot B.B. (1967). How long is the coast of Britain? Statistical self-similarity and fractional dimension. Science.

[35] Mandelbrot B.B. (1975). Stochastic models for the Earth’s relief, the shape and the fractal dimension of the coastlines, and the number-area rule for islands. Proc. Natl. Acad. Sci..

[36] Mandelbrot B.B. (1982). The Fractal Geometry of Nature.

[37] Peitgen H.-O., Juergens H., Saupe D. (2004). Chaos and Fractals.

[38] Peleg S., Naor J., Hartley R., Avnir D. (1984). Multiple resolution texture analysis and classification. IEEE Trans. Pattern Anal. Mach. Intell..

[39] Paskas M.P., Gavrovska A.M., Reljin N.B. Identification of fundamental heart sounds from PCG using blanket fractal dimension. Proceedings of the 8th Conference of the European Study Group on Cardiovascular Oscillations.

[40] Turner M.J., Blackledge J.M., Blackledge J.M., Evans A.K., Turner M.J. (2002). Analysis of the limitations of fractal dimension texture segmentation for image characterisation. Fractal Geometry: Mathematical Methods, Algorithms, Applications.

[41] Yadollahi A., Moussavi Z.M.K. (2006). A robust method for estimating respiratory flow using tracheal sounds entropy. IEEE Trans. Biomed. Eng..

[42] Que C.-L., Kolmaga C., Durand L.-G., Kelly S.M., Macklem P.T. (2002). Phonospirometry for noninvasive measurement of ventilation: Methodology and preliminary results. J. Appl. Physiol..

